# Design of online combinatorial auction mechanism for urban land transfer

**DOI:** 10.1371/journal.pone.0284775

**Published:** 2023-10-31

**Authors:** Yajuan Wang, Yan Zhang

**Affiliations:** 1 Management School, Wuhan University of Science and Technology, Wuhan, Hubei province, China; 2 Centre for Service Science and Engineering, Wuhan University of Science and Technology, Wuhan, Hubei province, China; Taylor’s University - Lakeside Campus: Taylor’s University, MALAYSIA

## Abstract

With the wide application of the "Tender, Auction, and Listing" system for land in China, it is of paramount importance to establish a sound land auction mechanism so as to avoid unreasonable land allocation. However, existing studies only focus on developers who are merely concerned about land combinations and offline auctions with low efficiency and high land unsold rate. To further improve land auction mechanism, we modeled the problem of land allocation as an online combinatorial auction. On this basis, we defined a land correction coefficient and designed an optimal online combinatorial auction mechanism that enabled developers to submit multiple combinations of land. Our designed mechanism proved to satisfy both incentive compatibility (IC) and individual rationality (IR), which can prevent developers from lying in the auction while winning higher revenue, reducing land unsold rate, increasing developer turnover rate and promoting the stability of land resource allocation rate. Therefore, online combinatorial auction mechanism, both applicable and efficient, is a practical solution for improving land auctions in China.

## Introduction

Ministry of Land and Resources of China has explicitly required that from 2007, all commercial construction land should be sold by “Tender, Auction, and Listing”, officially marketizing the allocation of land resources and making it an irreversible trend for the government to allocate land by means of public bidding [1]. In the context of land marketization, different allocation methods will lead to fluctuations in land price, which in turn will affect the government’s revenue [2]. As an important part of deepening supply-side reform, land marketization can help improve economic operating environment and promote regional technological innovation [3]. However, most of land in China is currently transferred to the highest bidder, which has produced a series of drawbacks. On the one hand, local governments are overly dependent on land finance, which directly promotes the continuous increase of land and housing prices [4]. On the other hand, there are barriers to entry in the land transfer market and resources are skewed toward a few large developers [5]. This leads to imperfect competition in the market, which in turn leads to a series of problems such as monopoly and asymmetric information [6]. Therefore, it is necessary to explore a method to attract more developers to get involved and reveal true information, so as to improve land transfer mechanism, promote fair competition, and ensure the stable development of urban land price and housing price.

Auction is considered the best method to reflect the scarcity and market value of land, generating higher expected revenue for sellers [7]. Land auctions have been studied in a number of domestic and international literature. For example, Chow and Ooi [8] used a special set of land sales data to confirm that using English auction would generate higher returns than first-price sealed auctions. Wu et al. [9] discussed the mechanism of state-owned land auctions and compared the efficiency of market and non-market mechanisms, confirming that non-market mechanism would not improve social welfare. Liu et al. [10] improved complementary sequential auctions using the characteristics of land quota transactions and designed an optimal pricing mechanism. However, all the studies in the above literature focus on traditional multi-item sequential auctions without taking into account the possible complementary or substitution relationships among lands and are prone to "exposure problems" [11].

To address this issue, Wang and Shao [12] designed an optimal combinatorial auction mechanism under the condition that developers have interdependent value. Combinatorial auctions allow bidders to bundle bids for a set of items, which reduces the costs caused by a large number of repeated auction efforts while fully expressing the bidders’ preferences [13]. As an effective form of auction, combinatorial auctions have been used in areas such as sale of radio spectrum license [14], B2B marketplaces [15], and multi-attribute procurement [16]. However, existing studies on land combinatorial auctions only focus on offline auctions with single-minded bidders and have some shortcomings. One is that if some land in the combination is auctioned off, other equally interested parties will be forced to pull out. Another is that offline auctions will not enable the government’s real-time decision making.

With the advent of the information age, online auction is widely used in various fields, such as eBay and emissions trading marketplaces [17, 18]. In online auction, the auctioneer needs to make decisions as information about types is revealed online without the knowledge of future developer types or future decision possibilities, which greatly increases the possibility of successful auction [19]. Currently, no relevant studies have applied online auctions to land transfer. Therefore, in order to reduce the limitation of single-minded bidders’ participation in auctions, as well as to satisfy real-time land transactions in a dynamic environment, this paper proposes an online combinational auction mechanism that allows developer to submit multiple combinations of land to fill the research gap.

On this basis, we propose an optimal online combinatorial auction mechanism in the context of land transfer. First, we assume that developers can arrive and depart the auction platform over time during the auction periods and submit multiple combinations of land. Under these assumptions, an optimal online combinatorial auction mechanism design model is constructed with the goal of maximizing the government’s revenue. Second, the model is solved to obtain the initial optimal payment and allocation. Then, in order to improve the initial allocation results, we define a land correction coefficient by considering land resource consumption rate, which leads to an improved optimal online combinatorial auction mechanism. Finally, we set up experiments to compare the performances of the improved mechanism proposed in this paper and offline combinatorial auction mechanism from certain perspectives.

The remainder of this paper is organized as follows. Section 2 introduces basic variables and hypothesis. Section 3 states the problem of mechanism design. Section 4 solves the problem, including reformulating the model and improving the model and implementation procedure of online mechanism. Section 5 is the experimental analysis. Section 6 summarizes the contribution of our work.

## Basic variables and hypotheses

Local government usually publishes a set of land transfer announcements on the official website, including land information and transfer method. In order for developers to fully express their preference for land, they must be allowed to submit multiple combinations. At the reported departure time, developers can immediately access to information on whether to get his or her submitted land combinations. On this basis, the following hypothetical conditions and variable descriptions are proposed:

1. There are *m* plots of land transferred by a local government at a finite, discrete time periods *T* = {1,2,…,*t*}. As each plot of land has different attributes, it is regarded as an indivisible heterogeneous item. Let *M* = {1,2,…,*m*} be the set of land for transfer, the number of plots transferred by the government is variable at period *t*(*t*∈*T*). In addition, the government needs to pay various fees when acquiring land. In order to make a profit, it is expected to sell the land based on the cost price, and we denote the reservation price of any plot *j*(*j*∈*M*) by *c*^*j*^.
2. Suppose there are *n* risk-neutral developers participating in the land auction, which is denoted as the set *N* = {1,2,…,*n*}, developers are allowed to submit multiple combinations to ensure fairness, and all the combinations submitted by all developers are denoted as set *S*. Developer *i*(*i*∈*N*) can submit *Q*_*i*_ combinations after arriving the auction platform, expressed as set $S_{i}=\{s_{i1},s_{i2},\ldots ,s_{iQ_{i}}\}$. Any combination, $s_{iq}=\{s_{iq}^{1},s_{iq}^{2},\ldots ,s_{iq}^{m}\}$, and any $s_{iq}^{j}\in (0,1)$, where $s_{iq}^{j}=0$ indicates that combination *s*_*iq*_ submitted by *i* is not interested in plot *j*, and $s_{iq}^{j}=1$ indicates the opposite. Given the possible substitution between different combinations, developers can be allocated at most one combination before departure.
3. Suppose *θ*_*i*_ is a valuation signal about *S*_*i*_ of developer *i*, related to private information such as their expected house price, other developers, as well as governments, only know that *θ*_*i*_ is distributed over the interval (*θ*_*i*_, $\bar{\theta_{i}}$) according to the distribution function *F*_*i*_(*θ*_*i*_) with associated density function *f*_*i*_(*θ*_*i*_). All developer’s signals are independent, and their distribution function *F*_*i*_(*θ*_*i*_) satisfies the monotonic hazard rate properties, i.e. $\lambda_{i}\equiv \frac{f_{i}(\theta_{i})}{1-F_{i}(\theta_{i})}$ increases with respect to *θ*_*i*_.
4. The type of developer *i* $\omega_{i}=(a_{i},d_{i},S_{i},\theta_{i})\in T\times T\times S\times (\underline{\theta_{i}},\bar{\theta_{i}}){\in \Omega }_{i}$, where *i* has arrival time *a*_*i*_ and departure time *d*_*i*_. *S*_*i*_ is the set of the land combinations that *i* is interested in, *θ*_*i*_ is the valuation signal about these combinations, and *Ω*_*i*_ is the collection of *i*’s all possible types. In the independent private value model, the valuation function of developer *i* can be expressed as a function of the valuation signal, denoted as *v*_*i*_(*θ*_*i*_). The valuation function *v*_*i*_(*θ*_*i*_) is a convex function that is continuous, differentiable, and strictly increasing about *θ*_*i*_. Thus, we can know that the valuation function *v*_*i*_(*θ*_*i*_) is second-order derivable on (*θ*_*i*_, $\bar{\theta_{i}}$), and $\frac{d^{2}v_{i}(\theta_{i})}{d{\theta_{i}}^{2}}\leq 0$.
  For example, when the government auctions four plots of land, a developer submits a type of [1,3,(1,1,0,0), (1,0,1,0), 100], which means that the developer arrives at the platform at *t* = 1 and departs the platform at *t* = 3. Then, the first land combination he is interested in is plots 1 and 2, and the second land combination is plots 1 and 3, with the valuation of one million Yuan for each combination.
5. Let *θ* = (*θ*_1_, *θ*_2_, *θ*_3_,…,*θ*_*n*_) be the set of valuation signals for all developers, and *θ*_−*i*_ = (*θ*_1_,…,*θ*_*i*−1_, *θ*_*i*+1_,…,*θ*_*n*_) be the set of valuation signals for all developers except *i* as well as $\Omega =\prod_{i\in N}\Omega_{i},\Omega_{-i}=\prod_{l\neq i,i,l\in N}\Omega_{l}$, then *ω* = (*ω*_1_, *ω*_2_, *ω*_3_,…,*ω*_*n*_) is a set of types for all developers, *ω*_−*i*_ = (*ω*_1_,…,*ω*_*i*−1_, *ω*_*i*+1_,…,*ω*_*n*_) is the set of types for all developers except *i*.
6. Rational developers may misreport their private information in order to maximize profits, including the time of arrival and departure, as well as valuation signals. We denote the type of any developer *i* may report as $\omega_{i}^{\prime },\omega_{i}^{\prime }=(a_{i}^{\prime },d_{i}^{\prime },S_{i},\theta_{i}^{\prime })|a_{i}\leq a_{i}^{\prime }\leq d_{i}^{\prime }\leq d_{i},S_{i}\epsilon S,\theta_{i}^{\prime }\in (\underline{\theta_{i}},\bar{\theta_{i}})\}$. Developer *i* cannot report an earlier arrival because he or she does not know about the mechanism until *a*_*i*_. In addition, developer *i* will not report his or her departure time $d_{i}^{\prime }$ later than *d*_*i*_, because once *i* obtains the land within the interval $\left[d_{i},d_{i}^{\prime }\right]$, then its valuation of the allocated land is 0, at which point it will be meaningless.

### Statement of the problem

The government wishes to design an auction mechanism to maximize his expected revenue. Therefore, according to the *Revelation Principle*, we limit the problem of the optimal online combination mechanism design to a direct mechanism in which "telling the truth" is an equilibrium strategy. We define mechanism {*X*, *P*} consisting of the allocation rule $X=\{x_{iq}^{t}(\omega )|t\epsilon T,i\in N,1\leq q\leq Q_{i}\}$ and the payment rule *P* = {*p*_*i*_(*ω*)|*iϵN*}, where $x_{iq}^{t}(\omega )\in \{0,1\}$ indicates whether developer *i* obtains the *q*th combination at period *t*, and that *p*_*i*_(*ω*)*ϵR* indicates the payment of *i*.

According to the hypothesis in the previous section, when developer *i* reports its true type *ω*_*i*_, then the ex post utility of developer *i* can be expressed as:

$$U_{i}(\omega_{i})=\sum_{q=1}^{Q_{i}}\sum_{t\epsilon T}v_{i}(\theta_{i})x_{iq}^{t}(\omega )-p_{i}(\omega )$$ (1)

Assuming that developer *i* misreports his type as $\omega_{i}^{\prime }$, and other developers who arrive the auction before *d*_*i*_ report their true type, then the ex post utility of developer *i* can be expressed as:

$$\sum_{q=1}^{Q_{i}}\sum_{t\epsilon T}v_{i}(\theta_{i})x_{iq}^{t}(\omega_{i}^{\prime },\omega_{-i})-p_{i}(\omega_{i}^{\prime },\omega_{-i})$$ (2)

In the online auction mechanism {*X*, *P*}, the expected revenue of the government can be written as:

$$R=Ε\{\sum_{i\in N}p_{i}(\omega )+\sum_{j=1}^{m}c^{j}[1-\sum_{i\in N}\sum_{q=1}^{Q_{i}}\sum_{t\epsilon T}s_{iq}^{j}x_{iq}^{t}(\omega )]\}$$ (3)

**Definition 1** We say that our mechanism is (ex post) *Incentive Compatibility* (IC) if, for any *iϵN*, when other developers report types truthfully, the developer who reports true type gets higher utility than those who misreport, the condition of IC can be expressed by:

$$U_{i}(\omega_{i})\geq \sum_{q=1}^{Q_{i}}\sum_{t\epsilon T}v_{i}(\theta_{i})x_{iq}^{t}(\omega_{i}^{\prime },\omega_{-i})-p_{i}(\omega_{i}^{\prime },\omega_{-i}),\forall i\epsilon N,{\omega_{i},\omega_{-i}\epsilon \omega ,\omega }_{i}^{\prime }$$ (4)

**Definition 2** We say that our mechanism is (ex post) *Individual Rational* (IR) if, when all developers report type truthfully, any developer *iϵN* will obtain non-negative utility regardless of whether he bids successfully or not, *U*_*i*_(*ω*_*i*_)≥0 i.e.

Now we can write down the problem faced by the government. The government wants to design an auction that maximizes expected revenue. We can then write the optimization model as:

$${}_{\left(X,P\right)}^{max}E\{\sum_{i\in N}p_{i}(\omega )+\sum_{j=1}^{m}c^{j}[1-\sum_{i\in N}\sum_{q=1}^{Q_{i}}\sum_{t\epsilon T}s_{iq}^{j}x_{iq}^{t}(\omega )]\}$$ (5)

Subject to IC, IR,

$$0\leq \sum_{i\in N}\sum_{q=1}^{Q_{i}}\sum_{t\epsilon T}s_{iq}^{j}x_{iq}^{t}(\omega )\leq 1,\forall j\epsilon M$$ (6)

$$\sum_{t\epsilon T}x_{iq}^{t}(\omega )\epsilon (0,1),\forall i\epsilon N,1\leq q\leq Q_{i}$$ (7)

where Formula (5) is the objective function of maximizing the government’s revenue, Formula (6) is the quantitative constraint, ensuring that no single plot is allocated to multi developers, and Formula (7) indicates any developer *i* will get all the land in a combination submitted by him or her, or nothing at all.

## Solution of the problem

### Reformulating the model

Aiming at the optimization model of the optimal mechanism design proposed in the previous section and following the idea of designing the optimal auction mechanism for offline single item proposed by Myerson (1981) [20], the government’s expected revenue function is first analyzed to separate the allocation and payment rules.

According to the envelope theorem, we can obtain the equivalent expressions of IC and IR as:

$$U_{i}(\omega_{i})=U_{i}(a_{i},d_{i},S_{i},\underline{\theta_{i}})+\sum_{q=1}^{Q_{i}}\sum_{t\epsilon T}\int_{\underline{\theta_{i}}}^{\theta_{i}}\frac{dv_{i}(y)}{dy}x_{iq}^{t}(a_{i},d_{i},S_{i},y,\omega_{-i})dy$$ (8)

$$\mathrm{If}\;\theta_{i}\geq \theta_{i}^{\prime },\;\sum_{t\epsilon T}x_{iq}^{t}(\omega_{i})\geq \sum_{t\epsilon T}x_{iq}^{t}(\omega_{i}^{\prime },\omega_{-i}),\forall i\epsilon N,\forall \theta_{i},\theta_{i}^{\prime }\in (\underline{\theta_{i}},\bar{\theta_{i}})$$ (9)

$$U_{i}(a_{i},d_{i},S_{i},\underline{\theta_{i}})\geq 0$$ (10)

Substituting Formula (8) into Formula (1), we yield an expression of *p*_*i*_(*ω*) as:

$$p_{i}(\omega )=\sum_{q=1}^{Q_{i}}\sum_{t\epsilon T}v_{i}(\theta_{i})x_{iq}^{t}(\omega )-U_{i}(a_{i},d_{i},S_{i},\underline{\theta_{i}})-\sum_{q=1}^{Q_{i}}\sum_{t\epsilon T}\int_{\underline{\theta_{i}}}^{\theta_{i}}\frac{dv_{i}(y)}{dy}x_{iq}^{t}(a_{i},d_{i},S_{i},y,\omega_{-i})dy$$ (11)

Substituting Formula (11) into the expression of the government’s expected revenue (Formula (3)) yields:

$$R=Ε\left\{\sum_{i\in N}\{\sum_{q=1}^{Q_{i}}\sum_{t\epsilon T}v_{i}(\theta_{i})x_{iq}^{t}(\omega )-U_{i}\left(a_{i},d_{i},S_{i},\underline{\theta_{i}}\right)-\sum_{q=1}^{Q_{i}}\sum_{t\epsilon T}\int_{\underline{\theta_{i}}}^{\theta_{i}}\frac{dv_{i}(y)}{dy}x_{iq}^{t}(a_{i},d_{i},S_{i},y,\omega_{-i})dy\}+\sum_{j=1}^{m}c^{j}[1-\sum_{i\in N}\sum_{q=1}^{Q_{i}}\sum_{t\epsilon T}s_{iq}^{j}x_{iq}^{t}(\omega )]\right\}$$ (12)

Interchanging the order of integration in the third term of the formula above, the calculation process is revised as:

$$\begin{array}{l} \sum_{q=1}^{Q_{i}}\sum_{t\epsilon T}\int_{\underline{\theta_{i}}}^{\theta_{i}}\frac{dv_{i}(y)}{dy}x_{iq}^{t}(a_{i},d_{i},S_{i},y,\omega_{-i})dy=\sum_{q=1}^{Q_{i}}\sum_{t\epsilon T}\int_{\underline{\theta_{i}}}^{\bar{\theta_{i}}}\int_{\underline{\theta_{i}}}^{\theta_{i}}\frac{dv_{i}(y)}{dy}x_{iq}^{t}(a_{i},d_{i},S_{i},y,\omega_{-i})f_{i}(\theta_{i})dyd\theta_{i}\\ \;=\sum_{q=1}^{Q_{i}}\sum_{t\epsilon T}\int_{\underline{\theta_{i}}}^{\bar{\theta_{i}}}\int_{\theta_{i}}^{\bar{\theta_{i}}}\frac{dv_{i}(y)}{dy}x_{iq}^{t}(a_{i},d_{i},S_{i},y,\omega_{-i})f_{i}(\theta_{i})dyd\theta_{i}\\ \;=\sum_{q=1}^{Q_{i}}\sum_{t\epsilon T}\int_{\underline{\theta_{i}}}^{\bar{\theta_{i}}}\frac{dv_{i}(\theta_{i})}{d\theta_{i}}\frac{1-F_{i}(\theta_{i})}{f_{i}(\theta_{i})}x_{iq}^{t}(\omega_{i})f_{i}(\theta_{i})d\theta_{i}\\ \;=\sum_{q=1}^{Q_{i}}\sum_{t\epsilon T}\frac{dv_{i}(\theta_{i})}{d\theta_{i}}\frac{1-F_{i}(\theta_{i})}{f_{i}(\theta_{i})}x_{iq}^{t}(\omega_{i}) \end{array}$$ (13)

Further substituting Formula (13) into Formula (12), the government’s expected revenue function can be written as:

$$R=Ε\left\{\sum_{i\in N}\{\sum_{q=1}^{Q_{i}}\sum_{t\epsilon T}v_{i}(\theta_{i})x_{iq}^{t}(\omega )-U_{i}(a_{i},d_{i},S_{i},\underline{\theta_{i}})-\sum_{q=1}^{Q_{i}}\sum_{t\epsilon T}\frac{dv_{i}(\theta_{i})}{d\theta_{i}}\frac{1-F_{i}(\theta_{i})}{f_{i}(\theta_{i})}x_{iq}^{t}(\omega_{i})\}+\sum_{j=1}^{m}c^{j}[1-\sum_{i\in N}\sum_{q=1}^{Q_{i}}\sum_{t\epsilon T}s_{iq}^{j}x_{iq}^{t}(\omega )]\right\}$$ (14)

Since the payment is only related to *U*_*i*_(*a*_*i*_, *d*_*i*_, *S*_*i*_, *θ*_*i*_) and is negatively correlated with government’s expected revenue, *U*_*i*_(*θ*_*i*_) should be the minimum value of 0. At this time, the government’s expected revenue function can be expressed as:

$$R=Ε\left\{\sum_{i\in N}\sum_{q=1}^{Q_{i}}\sum_{t\epsilon T}\left[v_{i}(\theta_{i})-\frac{dv_{i}(\theta_{i})}{d\theta_{i}}\frac{1-F_{i}(\theta_{i})}{f_{i}(\theta_{i})}-\sum_{j=1}^{m}c^{j}s_{iq}^{j}\right]x_{iq}^{t}(\omega )+\sum_{j=1}^{m}c^{j}\right\}$$ (15)

The payment becomes:

$$p_{i}(\omega )=\sum_{q=1}^{Q_{i}}\sum_{t\epsilon T}\left[v_{i}(\theta_{i})x_{iq}^{t}(\omega )-\sum_{q=1}^{Q_{i}}\int_{\underline{\theta_{i}}}^{\theta_{i}}\frac{dv_{i}(y)}{dy}x_{iq}^{t}(a_{i},d_{i},S_{i},y,\omega_{-i})dy\right]$$ (16)

Therefore, if there is an allocation $x_{iq}^{t}(\omega )$ which makes Formula (15) reach the maximum under the premise of satisfying Formulas (6) and (9), while the payment is given by the (16), then the mechanism {*X*, *P*} is an optimal online combinatorial auction mechanism.

### Improving the model

The mechanism described in previous subsection has two deficiencies, one of which is that the structure of (9) is complex, making it difficult to solve the allocation $x_{iq}^{t}(\omega )$ by calculation, and the other is that there will be a distinction between "popular" and "unpopular" due to the different properties of the land to be auctioned. "Popular" land is prone to fierce competition, resulting in premature auction, while "unpopular" land is prone to very few bidders, resulting in the failure to transfer at auction [21]. Therefore, it is necessary for the government to balance the goal of maximizing revenue and average land resource consumption rate in order to scientifically and reasonably allocate land resources.

Hence, this section will propose an improved mechanism {X*, P*} that relies only on valuation signals. The improved mechanism can maximize the government’s revenue while averaging the consumption rate of land resources to be transferred to reduce the land unsold rate.

To simplify the objective function, we define $\varphi (\theta_{i})=v_{i}(\theta_{i})-\frac{dv_{i}(\theta_{i})}{d\theta_{i}}\frac{1-F_{i}(\theta_{i})}{f_{i}(\theta_{i})}$ to be the virtual valuation of developers with valuation signal *θ*_*i*_, denoted as Formula (17). *φ*(*θ*_*i*_) can be understood as the contribution of the developer *i* to the government’s revenue when it reports its signal to be *θ*_*i*_, then Formula (15) can be re-written as:

$$R=Ε\left\{\sum_{i\in N}\sum_{q=1}^{Q_{i}}\sum_{t\epsilon T}\left[\varphi (\theta_{i})-\sum_{j=1}^{m}c^{j}s_{iq}^{j}\right]x_{iq}^{t}(\omega )+\sum_{j=1}^{m}c^{j}\right\}$$ (18)

Obviously, when the contribution of any developer is less than the sum of the reservation prices of the bidding combination, the government will not transfer these plots, otherwise, government will allocate the land to the developer with the highest contribution at period *t* subject to constraints (6) and (9). However, since the structure of (9) is unknown, it is still difficult to solve the allocation. Now, we need to prove that *φ*(*θ*_*i*_) is a monotone strictly increasing function of *θ*_*i*_ for every *i*∈*N*, in order to illustrate our mechanism design problem is *regular*. On this basis, we consider proposing a more specific land auction mechanism.

**Lemma 1.** ∀*i*∈*N*, $\theta_{i}\in (\underline{\theta_{i}},\bar{\theta_{i}}),$ *φ*(*θ*_*i*_) is strictly increasing about *θ*_*i*_, $\frac{d\varphi (\theta_{i})}{d\theta_{i}}>0$ i.e.

**Proof.** It is known from hypothesis 3 that $\frac{dv_{i}(\theta_{i})}{\partial \theta_{i}}>0$, $\frac{d^{2}v_{i}(S_{i},\theta_{i})}{d{\theta_{i}}^{2}}\leq 0$ and $\frac{d}{d\theta_{i}}\frac{1-F_{i}(\theta_{i})}{f_{i}(\theta_{i})}\leq 0$, then we can know that $\frac{d\varphi (\theta_{i})}{d\theta_{i}}>0$ and *φ*(*θ*_*i*_) is strictly increasing about *θ*_*i*_.

According to the model, the meaning of the relevant variables involved in the upcoming online combinatorial auction mechanism {X*, P*} is explained as follows:

1) $A^{t}=\{i|t\epsilon [a_{i},d_{i}],i\in N\}$ is the set of developers participating in the auction at period *t*, *W*^*t*^ is the set of developers who have successfully bid arriving at or before *t*, and *M*^*t*^ is the collection of land sold at *t*.
2) The correction coefficient *e*_*j*_ for plot *j* to correct the virtual valuation of combination *s*_*iq*_ submitted by the developer *i* is defined as:

$$e_{j}=\frac{\sum_{i\in A^{t}/W^{t-1}}\sum_{q=1}^{Q_{i}}{s_{iq}}^{j}}{\sum_{i\in A^{t}/W^{t-1}}\left|Q_{i}\right|}$$ (19)

where ∑*i*∈*A*^*t*^/*W*^*t*−1^|*Q*_*i*_| is the number of combinations submitted by all developers participating in the auction at period *t*.

Then, the revised virtual valuation of the combination *s*_*iq*_ submitted by the developer *i* is:

$$\varphi_{iq}(\theta_{i})=\frac{\varphi (\theta_{i})}{\sum_{j\epsilon M^{t}}s_{iq}^{j}e_{j}}$$ (20)

3) In the second section, we have presented that the reservation price of *j* is *c*^*j*^, then the reservation price of the combination *s*_*iq*_ submitted by developer *i* is:

$$c_{iq}=\sum_{j=1}^{m}c^{j}∙s_{iq}^{j}$$ (21)

4) For any land combination *s*_*iq*_ (1≤*q*≤*Q*_*i*_) submitted by developer *i* (*i*∈*A*^*t*^/*W*^*t*−1^) participating in the auction at *t*, if there is a land combination *s*_*lh*_ (1≤*h*≤*Q*_*l*_) submitted by developer *l* (*l*∈*A*^*t*^/*W*^*t*−1^) with $s_{iq}^{j}=s_{lh}^{j}=1$, then *s*_*lh*_ is a competing combination of *s*_*iq*_. Let $\varphi_{-i}^{t}=\{\varphi_{lh}(\theta_{l})|l\in A^{t}/W^{t-1},s_{iq}^{j}=s_{lh}^{j}=1,j\in M,1\leq h\leq Q_{l}\}$ be the set of revised virtual valuations of all competing combinations of *s*_*iq*_, then $max\varphi_{-i}^{t}$ be the maximum value in the set $\varphi_{-i}^{t}$

According to lemma 1 and Formula (20), in order to win the combination *s*_*iq*_, for every *i*∈*N*, *φ*_*iq*_ reaches maximum at any *tϵ*[*a*_*i*_, *d*_*i*_] when revealing true signal *θ*_*i*_. Thus, we define $\theta_{i}^{t}(\omega_{-i})$ as the lowest signal for which *i* can get from *s*_*iq*_ at *t*:

$$\theta_{iq}^{t}(\omega_{-i})=inf\left\{z_{i}^{t}|\varphi_{iq}(z_{i}^{t})\geq max\{\varphi_{-i}^{t},c_{iq}\},z_{i}^{t}\epsilon (\underline{\theta_{i}},\bar{\theta_{i}}),s_{iq}^{j}=s_{lh}^{j}=1,j\in M,i,l\epsilon A^{t}/W^{t-1}\right\}$$ (22)

Then, on the premise of $\varphi (\theta_{i})\geq c_{iq},\theta_{iq}(a_{i},d_{i},S_{i},\omega_{-i})=inf\left\{\theta_{iq}^{t}(\omega_{-i})|t\epsilon [a_{i},d_{i}]\right\}$ is defined as the lowest signal that the developer *i* can get from *s*_*iq*_ within [*a*_*i*_, *d*_*i*_]. If $v_{i}(\theta_{iq}(a_{i},d_{i},S_{i},\omega_{-i}))$ is less than the reservation price *c*_*iq*_, then the reservation price is paid. In a word, developer *i* should pay $max\{v_{i}(\theta_{iq}(a_{i},d_{i},S_{i},\omega_{-i})),c_{iq}\}$ after obtaining the combination *s*_*iq*_.

**Theorem 1**. If the allocation rule $x_{iq}^{t}(\omega )$ satisfies $x_{iq}^{t}(\omega )=\left\{ \begin{array}{c} 1,\theta_{i}>\theta_{iq}(a_{i},d_{i},S_{i},\omega_{-i})\\ 0,\theta_{i}\leq \theta_{iq}(a_{i},d_{i},S_{i},\omega_{-i}) \end{array}\right.$, and the payment rule *p*_*i*_(*ω*) satisfies $p_{i}(\omega )\left\{ \begin{array}{l} max\{v_{i}(\theta_{iq}(a_{i},d_{i},S_{i},\omega_{-i})),c_{iq}\},x_{iq}(\omega )=1\\ 0,other \end{array}\right.$, Then {X*, P*} is an improved optimal online combinatorial auction mechanism.

**Proof**. First, according to lemma 1, *φ*(*θ*_*i*_) increases with *θ*_*i*_. if $\theta_{i}\geq \theta_{i}^{\prime }$, then $\varphi (\theta_{i})\geq \varphi (\theta_{i}^{\prime })$. According to Formula (20), *φ*_*iq*_(*θ*_*i*_) also increases with *θ*_*i*_. Therefore, we can conclude that if $\theta_{i}\geq \theta_{i}^{\prime }$, then $\varphi_{iq}(\theta_{i})\geq \varphi_{iq}(\theta_{i}^{\prime })$. Furthermore, as defined by Formula (22) and *θ*_*iq*_(*a*_*i*_, *d*_*i*_, *S*_*i*_, *ω*_−*i*_), if any $\left[a_{i}^{\prime },d_{i}^{\prime }]\subseteq [a_{i},d_{i}\right]$, then $\theta_{iq}(a_{i},d_{i},S_{i},\omega_{-i})\leq \theta_{iq}(a_{i}^{\prime },d_{i}^{\prime },S_{i},\omega_{-i})$, then $\sum_{t\epsilon T}x_{iq}^{t}(\omega_{i})\geq \sum_{t\epsilon T}x_{iq}^{t}(\omega_{i}^{\prime },\omega_{-i})$ when $\theta_{i}\geq \theta_{i}^{\prime }$, so constraint (9) is true. Further, according to the allocation rules described in Formula (22) and theorem 1, it can be known that the quantitative constraint (6) is satisfied.

Second, after the introduction of the land correction coefficient *e*_*j*_, the initial virtual valuation of the land is revised. Formula (20) indicates that the more popular the land in all bid combinations, the lower the revised virtual valuation. Finally, according to Formula (18), the contribution of any combination needs to be greater than its reservation price. Thus, under the premise of introducing *e*_*j*_, the allocation rule in Theorem 1 is equivalent to the allocation maximized by Formula (15).

When $x_{iq}^{t}(\omega )=1$, there are two scenarios. If there is at least one competitive combination, payment can be calculated according to (16):

$$p_{i}(\omega )=\sum_{q=1}^{Q_{i}}\sum_{t\epsilon T}\left[v_{i}(\theta_{i})x_{iq}^{t}(\omega )-\sum_{q=1}^{Q_{i}}\int_{\theta_{iq}(a_{i},d_{i},S_{i},\omega_{-i})}^{\theta_{i}}\frac{dv_{i}(y)}{dy}x_{iq}^{t}(a_{i},d_{i},S_{i},y,\omega_{-i})dy\right]=v_{i}(\theta_{iq}(a_{i},d_{i},S_{i},\omega_{-i})).$$

If there is no competitive combination, developer *i* will report $v_{i}(\theta_{iq}^{t}(\omega_{-i}))=c_{iq}$, because the government will not transfer the land for an amount lower than the reservation price. Thus, $max\{v_{i}(\theta_{iq}(a_{i},d_{i},S_{i},\omega_{-i})),c_{iq}\}$ is equivalent to the payment rule in Theorem 1.

### The mechanism implementation procedures

The improved online combinatorial auction mechanism program is divided into three major steps, namely bidding transfer, running winner determination and calculating payment. The specific implementation steps are as follows:

### Bid transformation

 **Algorithm 1  *Bid-Trans***

**Input:**a) time *t*; b) types set *ω*; c)the reservation price set *C*; d)transferred land set at *t*

**Output:**The revised virtual bid set *φ*^*t*^;

1:**for** *i*∈*A*^*t*^/*W*^*t*−1^ **do**

2: **for** *s*_*iq*_^*j*^ = 1 **do**

3:        **if** *j∉M*^*t*^ **then**

4:           *S*_*i*_ = *S*_*i*_\{*S*_*iq*_}

5:        **else *Calc*** *c*_*iq*_, *φ*(*θ*_*i*_)

4:               **if** *c*_*iq*_>*φ*(*θ*_*i*_) **then**

5:                   *S*_*i*_ = *S*_*i*_\{*S*_*iq*_};

6:              **end if**

7:        **end if**

8: **end for**

9: **end for**

10: *φ*^*t*^ = ∅;

11: **for** *s*_*iq*_*ϵS*_*i*_ **do**

12:  ***Calc*** *e*_*j*_;

13: **end for**

14: **if** *e*_*j*_≠0 **then**

15: **for** *i*∈*A*^*t*^\*W*^*t*−1^ **do**

16:    **for** *s*_*iq*_*ϵS*_*i*_ **do**

17:      ***Calc*** *φ*_*iq*_(*θ*_*i*_);

18: *φ*^*t*^ = *φ*^*t*^∪{*φ*_*iq*_(*θ*_*i*_)};

19:    **end for**

20:  **end for**

21:**end if**

22:**Return** *φ*^*t*^;

### Winner determination

**Algorithm 2 *Cha-Alloc***

**Input:**a) time *t*; b)The revised virtual bid set *φ*^*t*^;c) the reservation price set *C*;

**Output:** *W*^*t*^

1:**for** *i*∈*A*^*t*^\*W*^*t*−1^ **do**

2: ***calc*** *φ*(*θ*_*i*_)

3:        **if** *s*_*iq*_*ϵS*_*i*_ **then**

4:          *φ*(*θ*_*i*_) = *φ*_*iq*_(*θ*_*i*_)

5:        **end if**

5:        *Calc* $max[\varphi_{iq}(\theta_{i})-\sum_{j=1}^{m}c^{j}s_{iq}^{j}]x_{iq}^{t}(\omega )$

6:         **while** $x_{lq}^{t}(\omega )=1$ **do**

7:         *W*^*t*^ = *W*^*t*^∪{*l*}

8: **end while**

10: **end for**

        **Return** *W*^*t*^

### Payment calculation

**Algorithm 3 *Pay-Calc***

**Input:** a)bidder *i*’s type *ω*_*i*_; b)the reservation price set *C*;

**Output:** Payment set *P*;

1: **for** $i\in A^{t}/W_{-l}^{t-1}$ **do**

2: **if** *s*_*ih*_*ϵS*_*i*_ and $s_{ih}^{j}=s_{lq}^{j}=1$ **then**

3:  ***Bid-Trans***

4:      **for** $\varphi_{-l}^{t}$ **do**

5:  $\theta_{lq}^{t}(\omega_{-l})=inf\{z_{l}^{t}|\varphi_{lq}(z_{l}^{t})\geq max\varphi_{-l}^{t}\}$;

4:  Calc $v_{l}(\theta_{lq}^{t}(\omega_{-l}))$;

5:      **else** $v_{l}(\theta_{lq}^{t}(\omega_{-l}))=c_{lq}$;

6:           **end for**

7:        **end if**

8: **end for**

9: $p_{l}(\omega )=\underset{t\in [a_{i},d_{i}]}{min}v_{l}(\theta_{lq}^{t}(\omega_{-l}))$

10: *P* = *P*∪*p*_*l*_;

**Return** *P*;

The above is a process of allocation and payment rules designed for improved online combinatorial auction mechanism {X*, P*}. In this mechanism, developer *i* can participate in the auction at *a*_*i*_≤*t*≤*d*_*i*_. If *i* succeeds in winning the land combination *s*_*iq*_, he or she should pay $max\{v_{i}(\theta_{iq}(a_{i},d_{i},S_{i},\omega_{-i})),c_{iq}\}$ at the reported departure time. If *i* is unsuccessful, then there is no need to pay such amount. When developer *i* obtains an arbitrary combination at a certain point in time, he or she will exit the platform after making payment at the reported departure time.

## Experimental analysis

Combined with the current situation of land transfer in China, we will verify the efficiency and applicability of our proposed auction mechanism in this section. In this experiment, it was assumed that the developers participating in the auction randomly arrived at the platform to submit multiple combinations. The existing land auction mechanism cannot satisfy the decision of developers to bid for multiple different combinations online. Therefore, we will compare the optimal offline combination auction mechanism proposed by Ülkü [22] and analyze the performance of {X*, P*} in terms of the government’s revenue, land unsold rate, and developer turnover rate, etc.

### Experimental setting

1. According to the land transfer announcement issued by China Land Market Network (https://www.landchina.com/) in early November 2021, a total of six plots of land for different purposes were transferred through an online auction in a city of Hubei Province. Based on the details of the announcement, the starting price of the auction set was regarded as the reservation price, and the reservation price of the six plots of land was respectively 1.971, 0.341, 0.86, 1.291, 0.347 and 2.3 (unit: 100 million Yuan).
2. The auction periods *T* was randomly taken in 5, 10, 15, 20, etc., and developers arrived at the platform randomly at any period during the auction, assuming that *d*−*a* of its stay was randomly generated in the interval [1, 5]. In addition, taking into account the city’s previous land auctions, it was assumed that any developer *i*(*iϵN*) could submit for three different combinations, *Q*_*i*_ = 3 i.e. and its signal about valuation *θ*_*i*_ was uniformly distributed on [5, 10].
3. To satisfy IR and the nature of the valuation function, the valuation function of any combination of the developer *i* was supposed to be *v*_*i*_ = *θ*_*i*_. In addition, the total number of developers participating in the auction *n* was randomly chosen among 5, 10, 15, 20, and 25. The six plots of land to be auctioned were randomly released at any period *T*.
4. Lingo software was used to obtain offline combination auction allocation results in the experiment, and payment result was calculated according to relevant formula. Using Matlab language, the bidding information of the mechanism {X*, P*} described in this paper was generated, and the results of the code running ten times were finally averaged as the final data.
5. In order to fully express the efficiency and applicability of the mechanism, the following four performance metrics were selected for comparative analysis:
  - Government’s revenue: $R=\sum_{i\in N}p_{i}(\omega )+\sum_{j=1}^{m}c^{j}[1-\sum_{i\in N}\sum_{q=1}^{Q_{i}}\sum_{t\epsilon T}s_{iq}^{j}x_{iq}^{t}(\omega )]$
  - Land unsold rate: $\frac{m-|G|}{m}$
  - Developer turnover rate: $\frac{\left|W\right|}{n}$
  - Land allocation rate: $\frac{\left|G\right|}{m}$

where *G* is the set of successfully transferred land and *W* is the set of developers who were successful in the auction.

First, in order to avoid "monopoly" in our land auction market, allow more developers to bid successfully and ensure a fair and equitable allocation of land resources, developer turnover rate was selected as an important indicator to measure developer’s demand satisfaction and transaction volume, thus reflecting the applicability of the proposed mechanism. Second, local governments depend on land finance, and the proposed mechanism aims to maximize the government’s revenue, which, therefore, was used as an indicator to determine whether this goal was achieved. Finally, evenly consumed land resources can make the mechanism more efficient and reduce problems such as transfer failure. Therefore, land unsold rate and land allocation rate were used as important metrics reflecting the efficiency, of which the former reflected the proportion of land to be auctioned that is not successfully sold and the latter reflected the speed and changing trend of land resource allocation of the mechanism proposed in this paper.

### Result analysis

According to the code running results, we selected *T* = 10, released three plots of land at t = 2, two plots of land at t = 3, and one plot of land at t = 5. We calculated the distribution and payment results of the proposed mechanism {X*, P*} and offline combinatorial auction mechanism respectively and obtained the following results:

S1 Fig compares land unsold rate. As can be seen from the figure, with the increase of developers, the overall land unsold rate of our mechanism fluctuated less significantly, remaining at around 33.33%, while that of offline combinatorial auction mechanism fluctuated slightly, reaching its maximum at 66.7%. In comparison, the land unsold rate of the mechanism proposed in this paper was significantly smaller than that of offline combinatorial auction mechanism, indicating that online auctions allowed developers to submit multiple combinations. In addition, land correction coefficient was designed to lower the risk of land abortion to a certain extent. The land is smoothly auctioned and can be delivered to the developer for construction as soon as possible, avoiding a series of management pressure on the government due to land lien.

S2 Fig compares developer turnover rate between the two mechanisms. From the figure, it can be seen that the developer turnover rate of our proposed mechanism was always higher than that of offline combinatorial auction mechanism, with a maximum of 66.7%, indicating that most of the developers participating in the auction were awarded the land. In addition, developer turnover rate under the two mechanisms gradually decreased with the increase in the number of developers due to the fact that land supply remains unchanged despite increasingly intense competition. It is clear that the proposed mechanism effectively avoids monopoly by individual developers and promotes the fairness and equity of land distribution.

S3 Fig compares the government’s revenue under the two mechanisms. It can be seen from the figure that the government’s revenue under the two mechanisms showed a downward trend with the increase in the number of developers. When there were 15 or less developers, the government’s revenue under the two mechanisms showed a downward trend with the increase in the number of developers. The reason lies in the high unsold rate of land in the offline combinatorial auction mechanism would cause the government to lose a large part of the revenue, which could be effectively avoided by the mechanism proposed in this paper.

S4 Fig shows a comparison of land allocation rate under the proposed mechanism. From the figure, it can be seen that land allocation rate firstly increased and then leveled off over time, remaining constant at t = 4 or t = 5. In addition, land allocation rate fluctuated slightly with the increase in the number of developers, but with stable performance as a whole. Thus, it could be concluded that land allocation rate in the online auction mechanism was not greatly affected by the number of participants in the auction but by land supply.

In summary, the optimal land online combinatorial auction mechanism proposed in this paper was superior to the optimal offline combinatorial auction mechanism in terms of the government’s revenue, land unsold rate, developer turnover rate, and the stability of land resource allocation rate. In addition, land resource allocation is also affected by land supply, so the government needs to ensure sufficient land supply in order to avoid too many rejected bids from developers.

## Conclusion

In order to improve the efficiency of land transfer and increase the government’s revenue, we designed an online combination auction mechanism in the context of state-owned land auctions and experimentally verified its feasibility. Compared with previous studies on land auction mechanisms, the proposed optimal online combinatorial auction mechanism has made the following contributions:

1. Developers can arrive and depart the auction platform at any period during the government-imposed auction periods, which reduces time and space constraints and thus time and opportunity costs for both parties. At the same time, the government is required to make a decision at each period to improve resource allocation efficiency and allow the allocated land to be delivered for construction as soon as possible.
2. Taking into account the possible complementarity and substitution of different combinations, our mechanism allows developers to submit multiple combinations of land that can fully express the developer’s preferences, which makes auctions much simpler and effectively avoids “exposure problem” particularly when there are many plots of land to be transferred.
3. On the basis of maximizing the government’s revenue, a land correction coefficient was proposed to obtain an improved land auction mechanism. According to the experimental results, the proposed mechanism can further reduce land unsold rate, increase developer turnover rate and promote the stability of land resource allocation rate while promoting the fair distribution and avoiding "monopoly" by individual developers.
4. The theoretical analysis proves that the auction mechanism proposed in this paper satisfies incentive compatibility and individual rationality, i.e. while attracting more developers to participate in the auction, the proposed mechanism can motivate them to "tell the truth", which helps the government to allocate land according to their true valuation and gain more revenue.

## Supporting information

S1 Fig Comparison of land unsold rate under the two mechanisms. (TIF)

S2 Fig Comparison of developer turnover rate under the two mechanisms. (TIF)

S3 Fig Comparison of the government’s revenue under the two mechanisms. (TIF)

S4 Fig Land allocation rate of the proposed mechanism. (TIF)

S5 Fig (TIF)

S1 Data (ZIP)
