# Supplementary figures and images for: Design of online combinatorial auction mechanism for urban land transfer
Source: PLoS One. 2023 Oct 31;18(10):e0284775. doi: 10.1371/journal.pone.0284775 (PMC10617715; doi:10.1371/journal.pone.0284775)

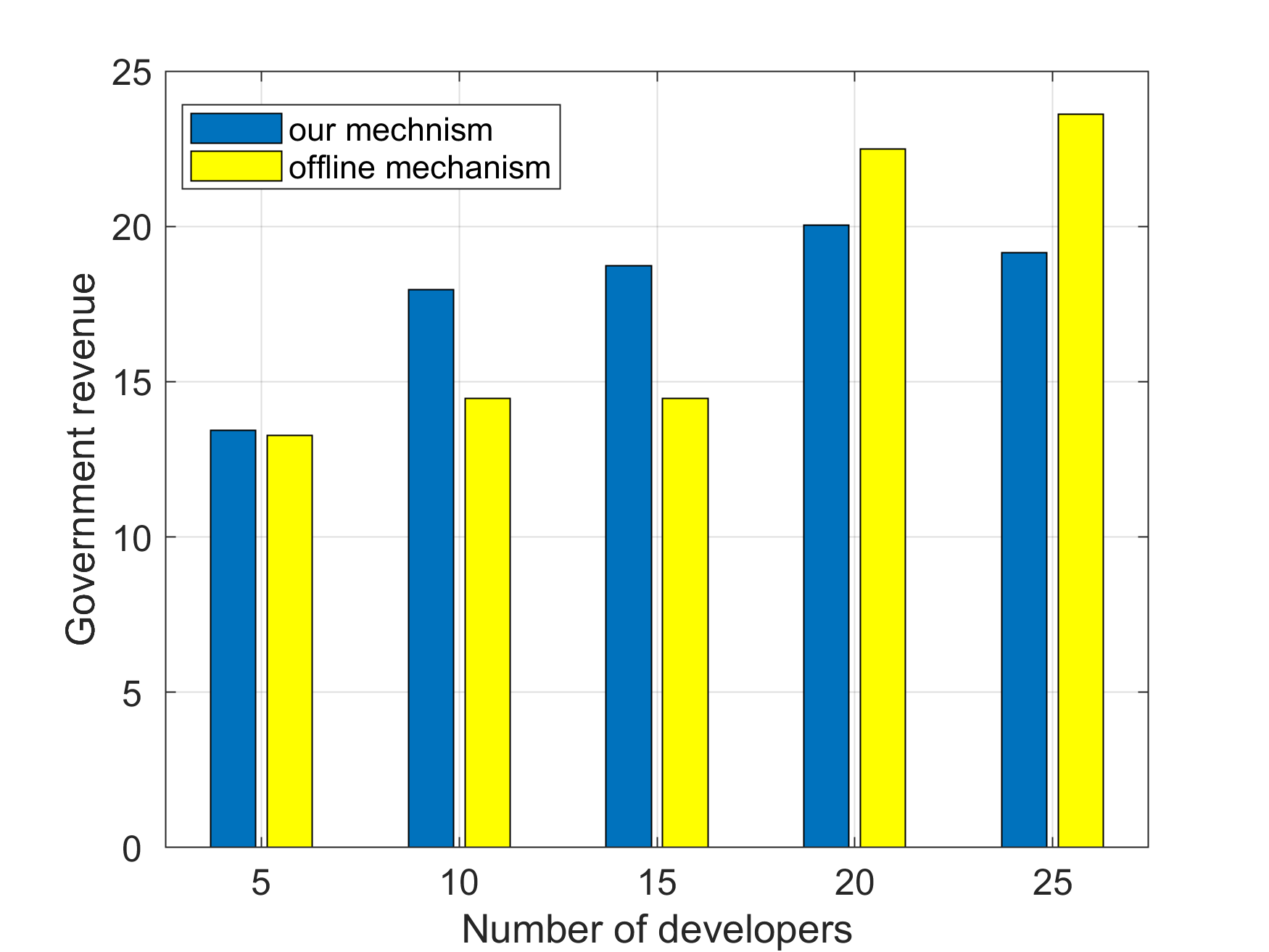

Supplement: S1 Fig — (TIF) [file pone.0284775.s001.tif]

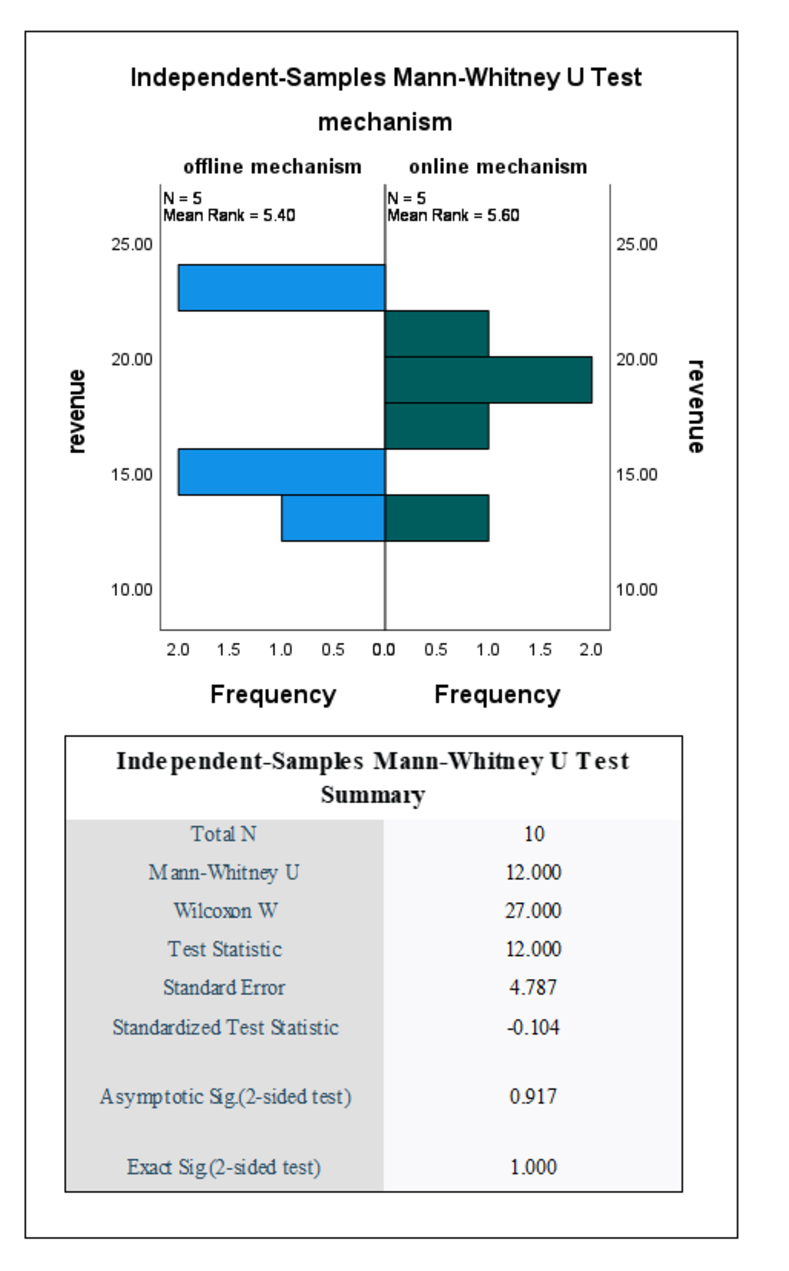

Supplement: S2 Fig — (TIF) [file pone.0284775.s002.tif]

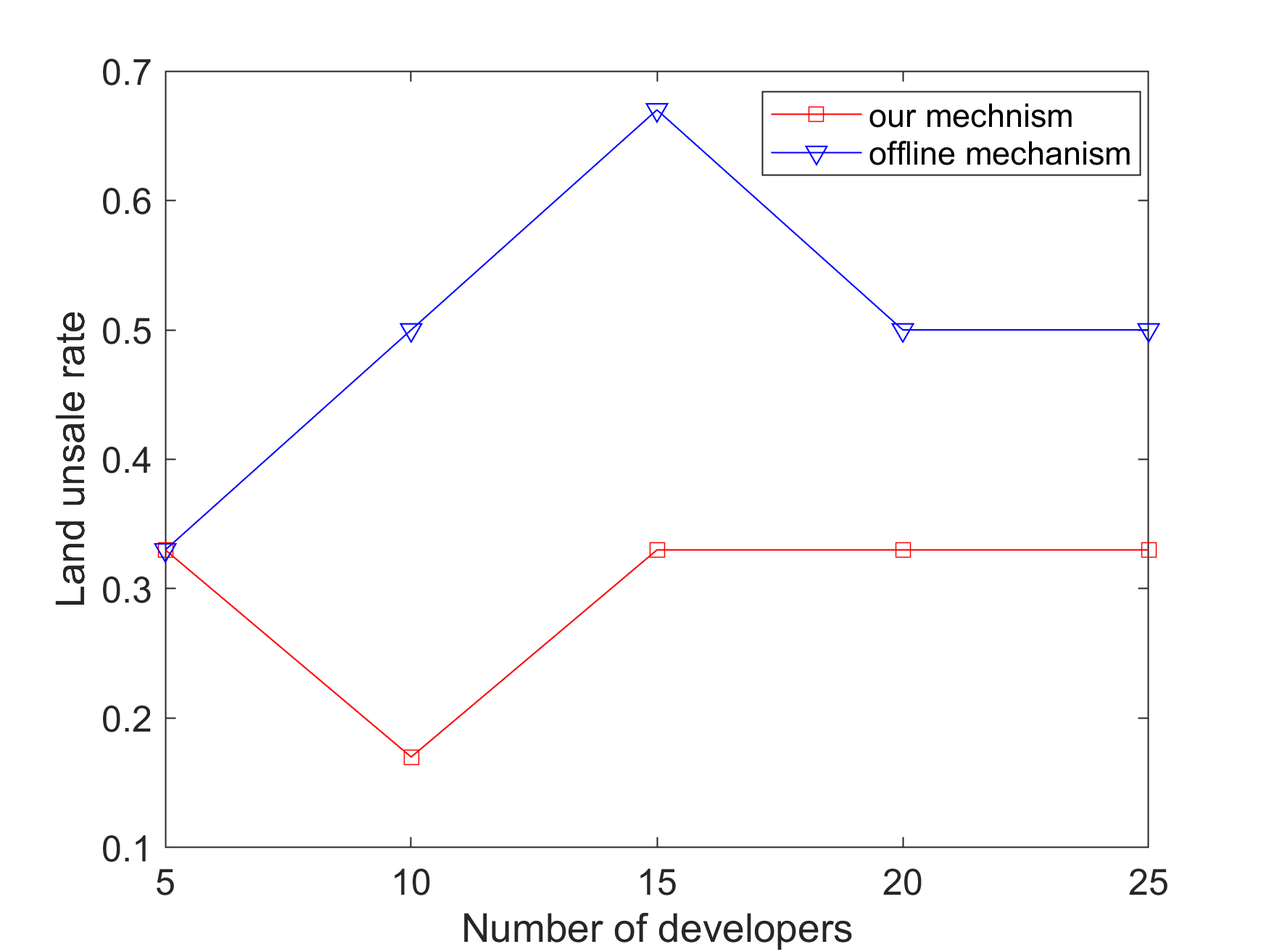

Supplement: S3 Fig — (TIF) [file pone.0284775.s003.tif]

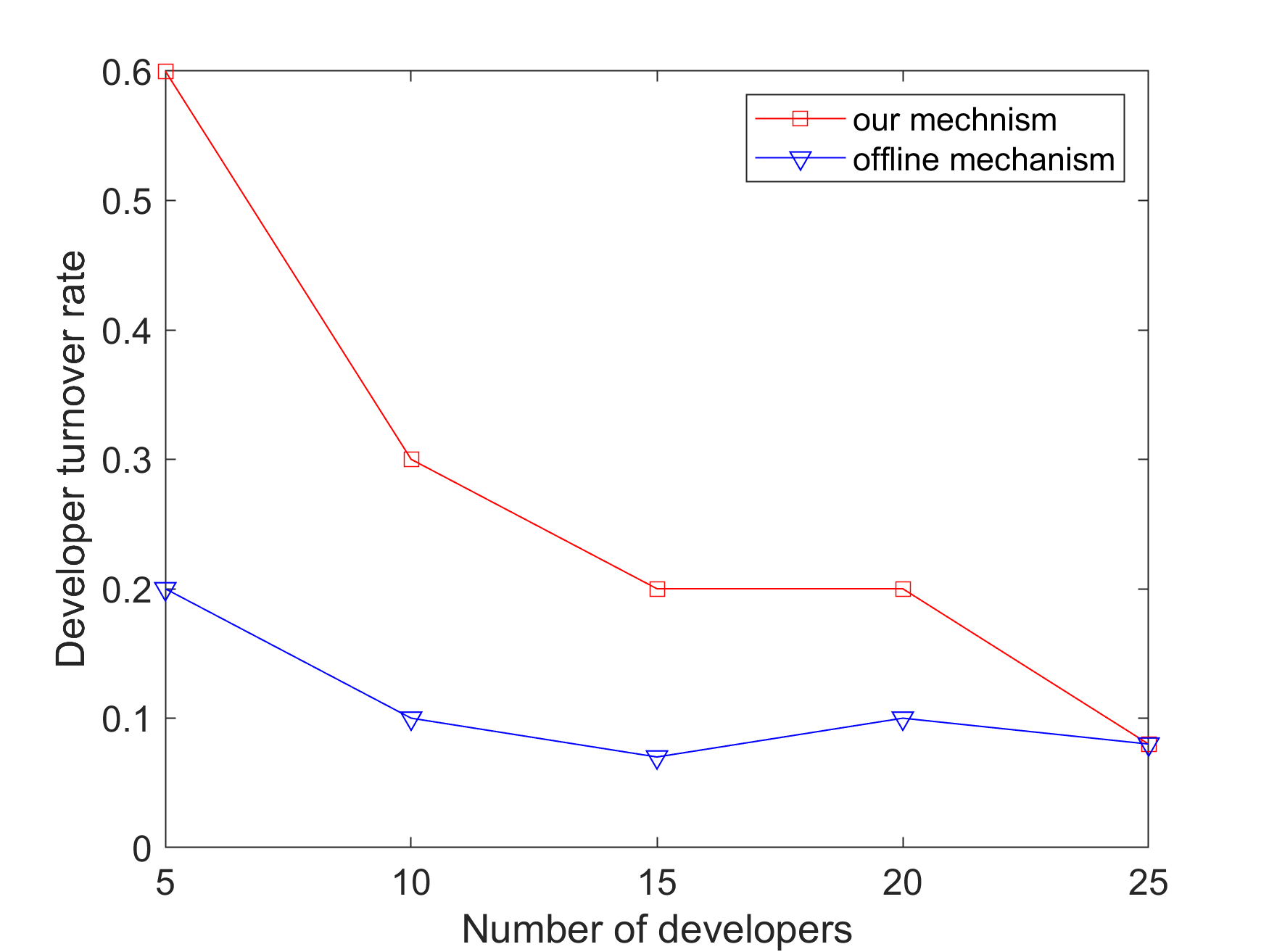

Supplement: S4 Fig — (TIF) [file pone.0284775.s004.tif]

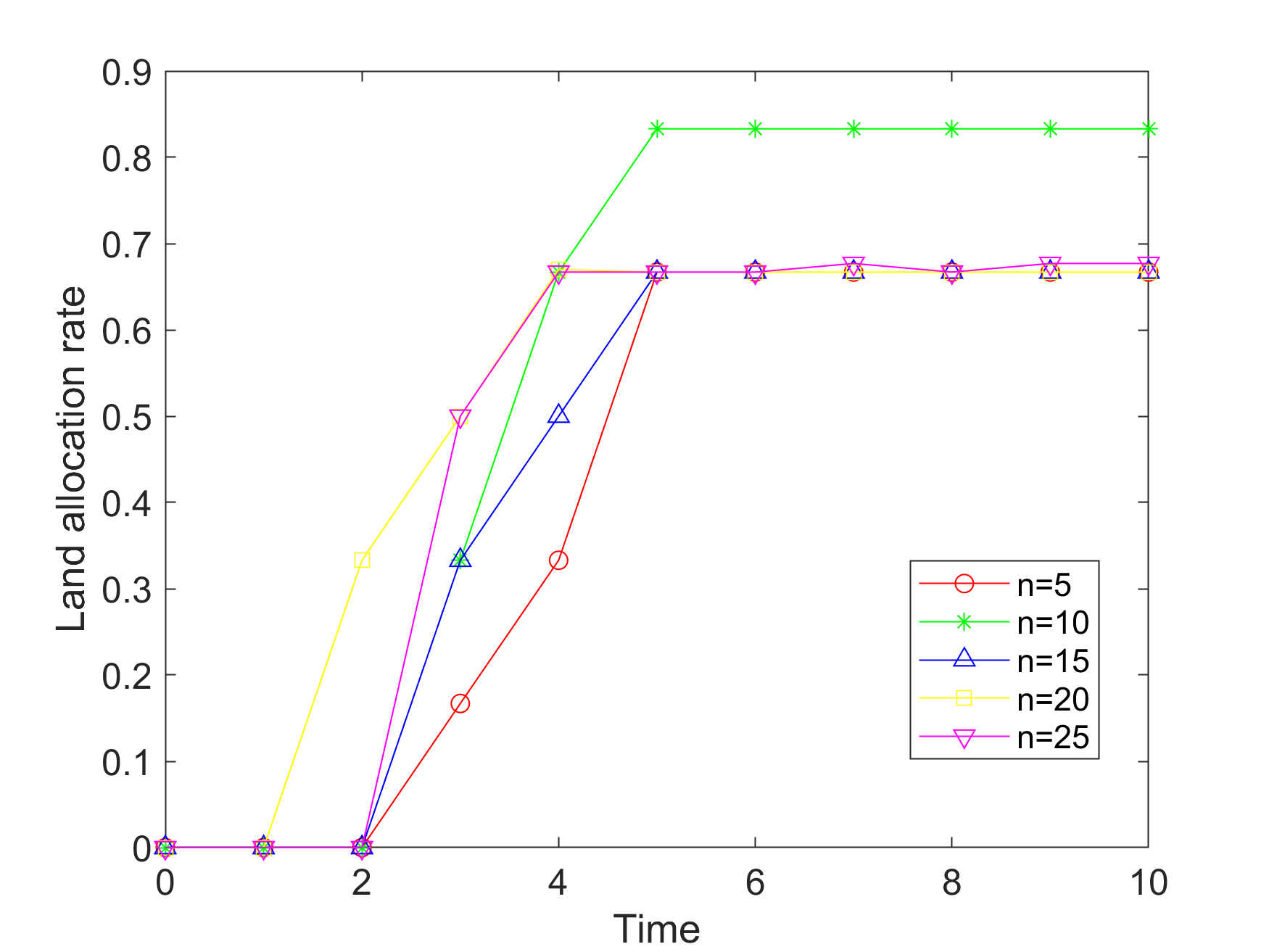

Supplement: S5 Fig — (TIF) [file pone.0284775.s005.tif]
